# The effects of post-activation performance enhancement and different warm-up protocols on swim start performance

**DOI:** 10.1038/s41598-022-13003-9

**Published:** 2022-05-31

**Authors:** Marko Đurović, Nikola Stojanović, Nenad Stojiljković, Dajana Karaula, Tomislav Okičić

**Affiliations:** 1 Faculty of Sport and Physical Education, University of Niš, Niš, Serbia; 2 Faculty of Kinesiology, Department of Sport, University of Zagreb, Zagreb, Croatia

**Keywords:** Physiology, Medical research

## Abstract

This study aimed to examine the effects of post-activation performance enhancement (PAPE) on swim start performance and lower body power performance after different warm-up protocols. Ten male national-level swimmers performed three different warm-ups: (i) a swim-specific warm-up (SW, control protocol); (ii) PAPE (an experimental protocol); and (iii) SW followed by PAPE (SW + PAPE, an experimental protocol). PAPE consisted of performing three series of 5 drop jumps. A repeated-measures ANOVA showed significant differences between the protocols in the swim start performance (F = 8.89; P < 0.001) and countermovement jump (F = 2.22; P = 0.047). SW + PAPE induced greater improvements in swim start time to 15 m (ES = − 0.47, P = 0.017) and entry time (ES = − 1.83, P < 0.001), the countermovement jump reactive strength index modified (ES = − 1.83, P < 0.001), eccentric rate of force development (ES = 0.69, P = 0.047), and index of explosive strength (ES = 0.94, P = 0.005) compared to SW. The current findings of this study indicate that the drop jump PAPE protocol, in addition to SW, is an effective tool because it could improve athletes' capacity for a more efficient swim start and their countermovement jump performance. Furthermore, the results of this study indicate that PAPE induced by drop jumps could be time-efficient and practically applicable in facilities with limited resources.

## Introduction

Swim start performance is an essential element of the swimming race, especially in 50-m competitions at the international level. It has been estimated that swim start performance can contribute up to 30 per cent of the total time of the 50-m race^1^. For example, at the Olympic Games (Tokyo, 2020), the men's 50-m freestyle gold medal was won with a time of 21.07 s, while the swimmer ranked eighth achieved a time of 21.79 s, which is a difference of 0.72 s and represents only 2.92% of winner’s total race time (www.fina.org). Hypothetically, it can be assumed that the time lost at the start can drastically affect the order in the final ranking. The swim start should include a fast reaction time, vertical and horizontal force off the block, high take-off velocity, and low hydrodynamic drag during entry and underwater gliding^2,3^. FINA rules set limits to the underwater starting distance up to 15 m, and this distance is now universally used to measure the total starting time. Swimmers who produce a greater force on the starting block produce greater flight and entry velocities and will be able to maintain a higher entry velocity, which could lead to a better start performance^4^. Lower body muscle power is one of the critical components of an efficiently performed swimming start and can play a significant role in performance improvement^5^. Therefore, warm-up strategies could be a significant contributor to performance. It is necessary to apply adequate warm-up protocols to prepare competitors for a 50-m race more efficiently.

Post-activation performance enhancement (PAPE) has been described as a very effective method for maximal excitation of the neuromuscular apparatus^6–9^, which can significantly contribute to the performance of movements requiring maximal voluntary effort as the swim start. Several studies have shown that PAPE induced by submaximal and maximal external loads, such as the back squat^10^, Yo-Yo squat^11^, lunge^11,12^, eccentric flywheel^12,14^, resistive power rack^15^, could be beneficial to the swim start performance. Conversely, some resistance protocols like hand paddles and parachutes^16^ and pull-ups^13^ did not improve swim start performance. Although some resistance protocols positively influenced the swim start performance, it is questionable whether any of them is sufficiently advantageous and practically applicable in actual competition.

In current practice, maximal voluntary effort activities, such as PAPE, have found their application in the warm-up protocol of swimmers^10,11,13,14,17^. However, a swim-specific warm-up could take place several hours before the swimming race. Therefore, one can assume that PAPE induced by submaximal and maximal external loads could be a very beneficial approach. The problem that frequently emerges in competitions is that there are no real opportunities to manage such protocols, which can be time-consuming^18^. In addition, recovery after external stress induced by heavy resistance can take up to 8 min or more^10,19^.

Moreover, the literature is still not sufficiently consistent on how fatigue during heavy resistance PAPE protocols may decay performance. The required recovery after PAPE stimuli has been interpreted differently, from 3 to 10 min^20,21^. Conversely, a recent meta-analysis^22^ emphasised that sufficient recovery occurs after 3–7 min in trained athletes. However, undeveloped athletes may need more time to recover from heavy resistance protocols^20,21^, which could negatively impact performance.

On the other hand, there is evidence that plyometrics can stimulate the neuromuscular apparatus as much as a heavy resistance^23^. Therefore, we assumed that plyometrics is the beneficial approach in which exercises such as drop jumps could be implemented^24–26^. Because of its simplicity does not require much space or any complex equipment, and if implemented with a proficient technique, it could be safe and time-efficient. Moreover, when properly dosed, plyometrics can cause sufficient stimulation without leading to neuromuscular fatigue, with a recovery of only 1–5 min^27^, which could be a beneficial approach just before an event. To our knowledge, only one study incorporated drop jumps in the warm-up protocol of swimmers^28^.

The present study relied on three different warm-up protocols, swim-specific (SW), PAPE, and swim-specific with PAPE (SW + PAPE), to evaluate whether a specific warm-up protocol affects the swim start performance. Therefore, this study aimed to examine the effect of PAPE on swim start performance after different warm-up protocols.

## Results

After the initial management of the raw data in MATLAB, they were exported to a statistical program to calculate basic descriptive parameters (mean and standard error). The Kolmogorov–Smirnov test (0.11–0.23) showed that the results were normally distributed^29^, and parametric statistical procedures were applied.

### Swim start performance

**Figure 1:**
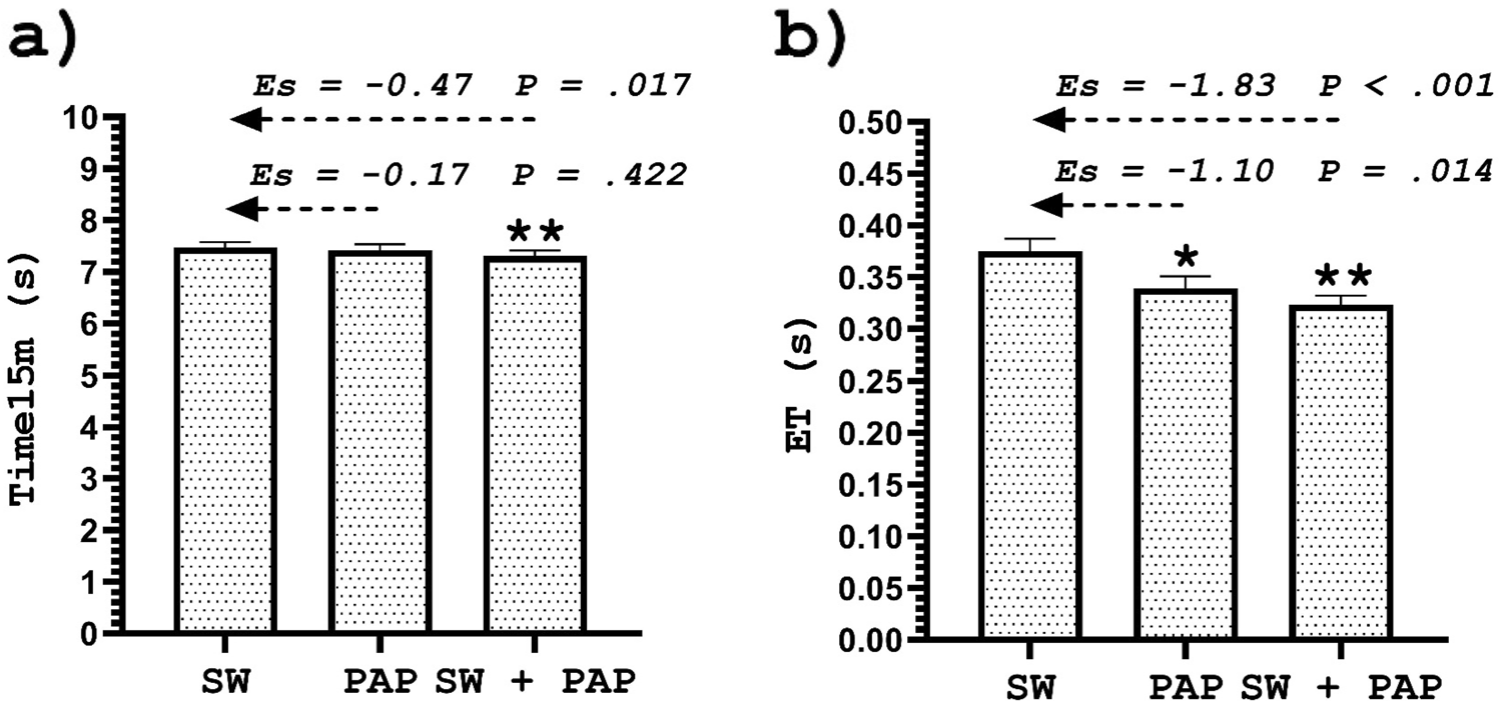
Swim start at 15 m (**a**) and entry time (**b**) after a swim-specific warm-up (SW), PAPE, and swim-specific warm-up with PAPE stimulus (SW + PAPE). *Indicates a significant difference between SW and PAPE. **Indicates a significant difference between SW and SW + PAPE. *ES* effect size. *P* p-value. The results are plotted as mean ± SE.

The swim start performance is shown in Table 1 and Fig. 1. There was a significant difference in the swim start performance (F = 8.89; P < 0.001) between the protocols. A significant increase was observed in swim time at 15 m (T15m) in SW + PAPE (ES = − 0.47, P = 0.017), but not in PAPE (ES = − 0.17, P = 0.422) compared to SW. Furthermore, a significant increase was observed in entry time (ET) in SW + PAPE (ES = − 1.83, P = 0.001) and the PAPE protocol (ES = − 1.10, P = 0.014) compared to SW (see Table 1 and Fig. 1). Improvements in the swim start performance were likely beneficial after SW + PAPE (0.1% harmful, 5.3% trivial, 94.6% beneficial), and unclear after PAPE (6.2% harmful, 42.8% trivial, 51.0% beneficial). Finally, reductions in ET were most likely beneficial after SW + PAPE (0.0% harmful, 0.1% trivial, 99.9% beneficial), and very likely beneficial after PAPE (0.0% harmful, 0.1% trivial, 99.9% beneficial).

**Table 1:** The CMJ and 15 m swim-start performance after SW, PAPE, and SW + PAPE.

Variables	SW	PAPE	SW + PAPE	Δ Change SW vs PAPE (%)	Δ Change SW vs SW + PAPE (%)
JH (m)	0.320 ± 0.02	0.332 ± 0.02	0.327 ± 0.01	3.79	2.47
RSImod	0.36 ± 0.02	0.41 ± 0.02	0.40 ± 0.02	11.66 *	10.54 **
PP (W kg^−1^)	46.03 ± 1.64	47.48 ± 1.80	46.56 ± 1.55	3.15	1.15
ERFD (N s^−1^ kg^−1^)	66.75 ± 4.61	72.31 ± 3.17	75.31 ± 5.39	8.34	12.83 **
IES	41.81 ± 1.64	45.88 ± 1.92	47.01 ± 1.81	9.73 *	12.43 **
T15m (s)	7.47 ± 0.10	7.41 ± 0.12	7.31 ± 0.11	0.84	2.31 **
ET (s)	0.38 ± 0.12	0.34 ± 0.01	0.32 ± 0.01	10.53 *	15.79 **

The data are reported as mean ± SE.

Adjustment for multiple comparisons: Bonferroni.

*JH* jump height, *RSImod* the reactive strength index modified, *PP* peak power, *ERFD* the eccentric rate of force development, *IES* the index of explosive strength, *T15m* swimming start corresponding to 15 m, *ET* entry time.

*Indicates a significant difference between the swim-specific warm-up (SW) and PAPE.

**Indicates a significant difference between the swim-specific warm-up (SW) and the swim-specific warm-up with PAPE (SW + PAPE).

### Countermovement jump (CMJ) performance

**Figure 2:**
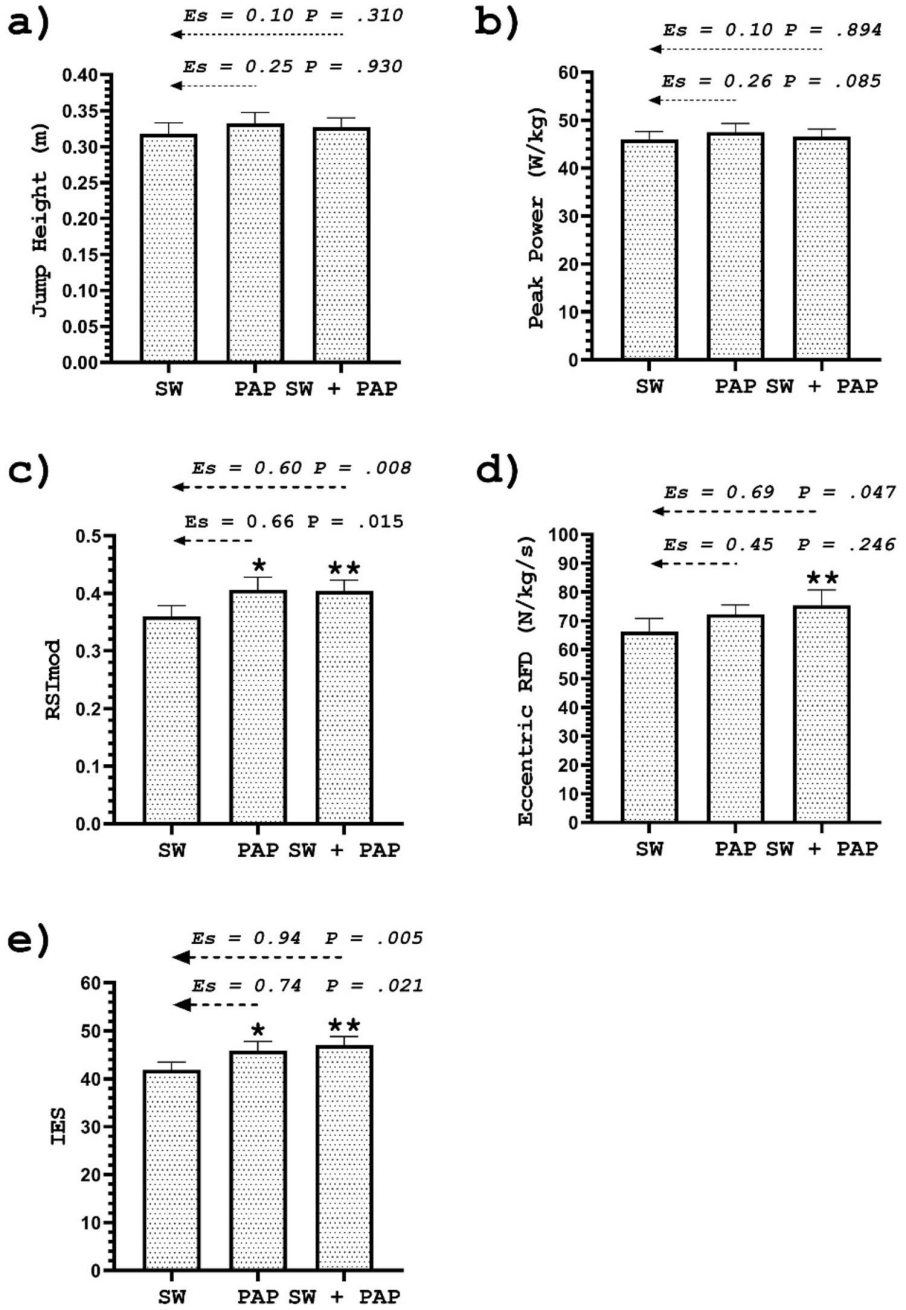
Jump height (**a**), peak power (**b**), RSImod (**c**), eccentric RFD (**d**), IES (**e**) after a swim-specific warm-up (SW), PAPE, and swim-specific warm-up with PAPE (SW + PAPE). *Indicates a significant difference between the SW and PAPE. **Indicates a significant difference between SW and SW + PAPE. *ES* effect size. *P-* p-value. The results are plotted as mean ± SE.

The results showed a significant mean difference between the protocols (F = 2.22; P = 0.047). A significant increase was observed in the reactive strength index modified (RSImod), and index of explosive strength (IES) in both PAPE and SW + PAPE compared to SW (see Table 1 and Fig. 2). Moreover, there was a significant increase in the eccentric rate of force development (ERFD) in SW + PAPE but not in PAPE compared to SW. Improvements in performance after PAPE and SW + PAPE were greater and very likely beneficial for the RSImod (0.1% harmful, 3.1% trivial, 96.8% beneficial, and 0% harmful, 2.4% trivial, 97.6% beneficial, respectively), IES (0.3% harmful, 3.5% trivial, 96.2% beneficial, and 0% harmful, 1.0% trivial, 99.0% beneficial, respectively), and likely beneficial for the ERFD in SW + PAPE (1% harmful, 54.2% trivial, 44.8% beneficial). Improvements in the ERFD after PAPE were unclear. There was no significant difference in jump height (JH) and peak power (PP) between protocols. However, performance improvements were small but possibly beneficial after PAPE and SW + PAPE for JH (0.3% harmful, 34.1% trivial, 65.6% beneficial, and 1.9% harmful, 54.1% trivial, 44% beneficial, respectively). Improvements in PP after PAPE were possibly beneficial (0.4% harmful, 28.4% trivial, and 71.2% beneficial) and unclear (35.6% harmful, 18.5% trivial, and 45.9% beneficial) after SW + PAPE.

**Figure 3:**
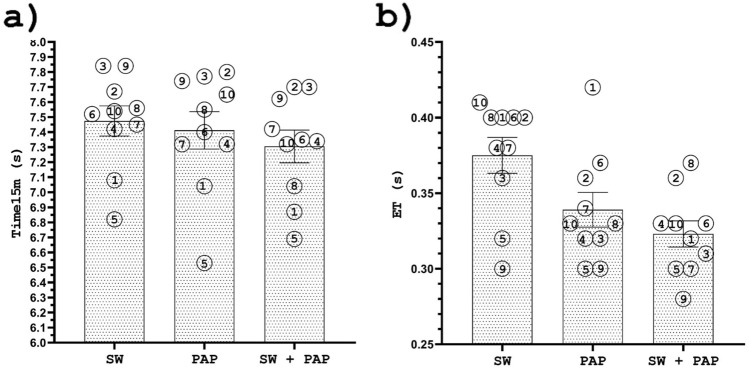
Individual responses for the swim start at 15 m (**a**) and entry time (**b**) after a swim-specific warm-up (SW), PAPE, and swim-specific warm-up with PAPE (SW + PAPE). The numbers indicate the athletes’ IDs. The results are plotted as mean ± SE.

Individual responses to different warm-up protocols for JH and PP are graphically presented in Fig. 3.

**Figure 4:**
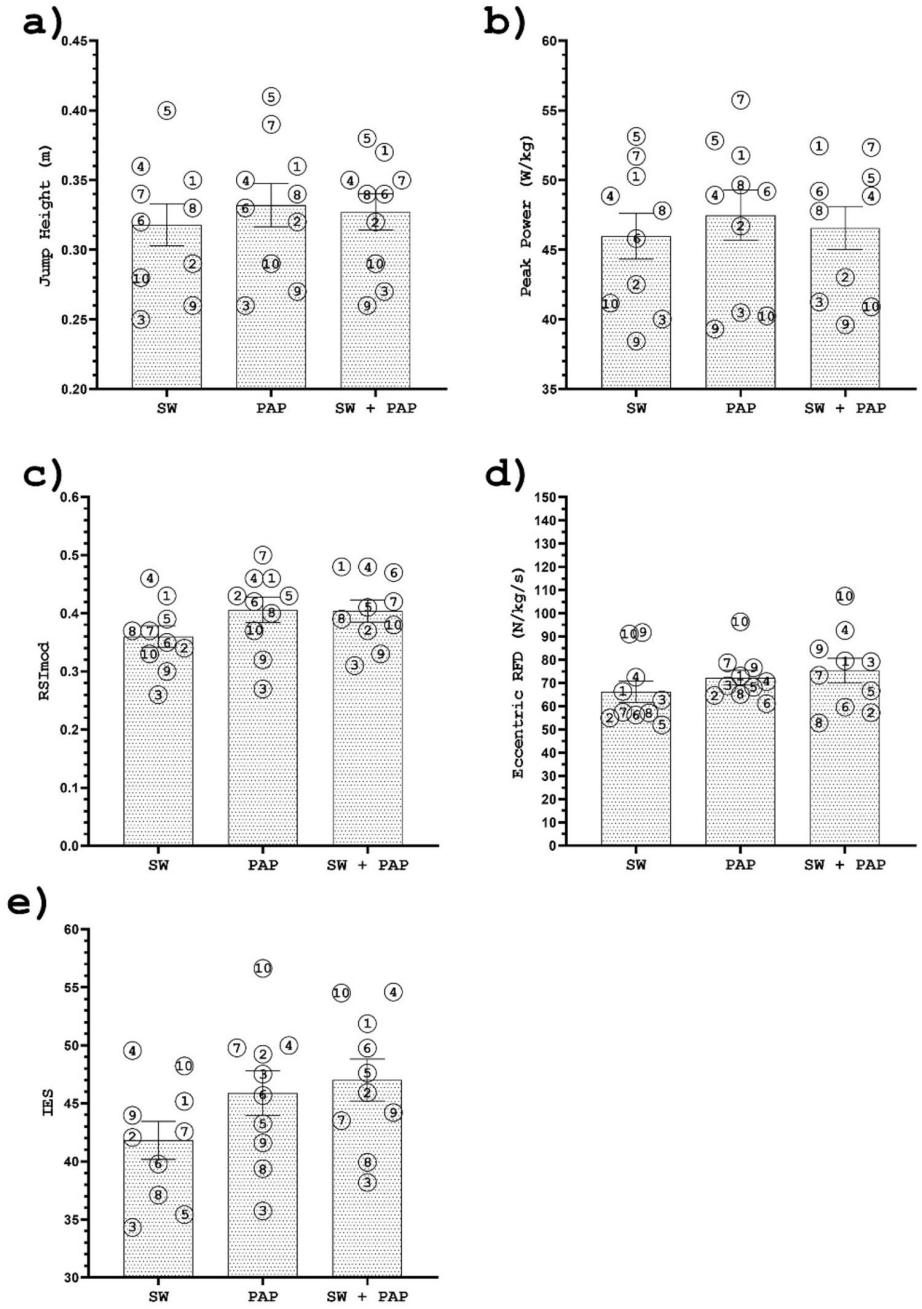
Individual responses for the jump height (**a**); peak power (**b**); the reactive strength index (**c**); eccentric RFD (**d**); and IES (**e**) after a swim-specific warm-up (SW), PAPE, and swim-specific warm-up with PAPE (SW + PAPE). The numbers indicate the athletes' IDs. The results are plotted as mean ± SE.

Individual responses to different warm-up protocols for T15m and ET are graphically presented in Fig. 4.

## Discussion

This study aimed to explore the effects of PAPE induced by drop jump performance in addition to a swim-specific warm-up on swim start performance. Additionally, we evaluated the CMJ performance following different warm-up protocols. The findings from the present study suggest that JH and PP did not significantly improve following experimental protocols compared to the control treatment. However, T15m, RSImod, ERFD, and IES may be positively influenced by implementing PAPE in addition to swim-specific warm-up, albeit to a moderate degree and a greater degree for ET.

The magnitude of improvement in the CMJ height was 3.79 and 2.49% for PAPE and SW + PAPE, respectively. However, although the results obtained were not statistically significant, the improvements were possibly beneficial and were similar to a previous study that employed a heavy resistance PAPE protocol in swimmers^10^. Several studies^23–25,30^ reported similar or slightly higher improvements induced by different drop jump protocols. Our athletes had previous experience in overall jumping performance; therefore, drop jumps from a 40 cm platform did not induce more significant improvements in the CMJ jump height. This statement could be supported by the main findings of Hilfiker et al.^25^, that athletes with some experience in jumping need more stimuli to improve their performance.

**Figure 5:**
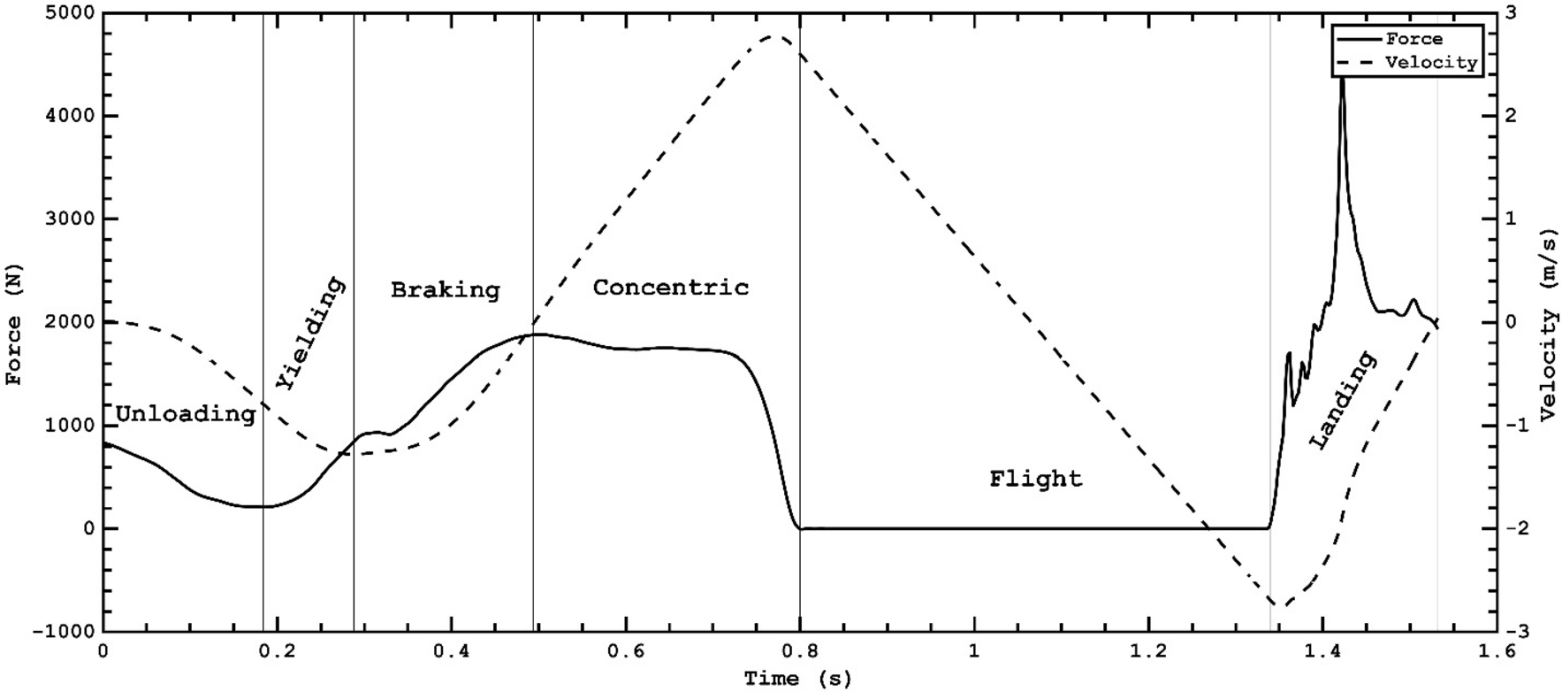
Phases of CMJ.

PP was positive but not significantly altered after specific protocols (3.15 and 1.15%, respectively), which is by a similar study (2.7%)^28^. Although plyometrics are less fatiguing and require only 1–5 min to recover^27^, younger athletes could encounter some fatigue when performing multiple sets of drop jumps^24^. Chen et al.^24^ reported that 3–5 repetitions of drop jumps could benefit efficient performance. However, PP could not fully explain the true nature of the jumping performance. Temporal strategic parameters should be taken into account when assessing overall performance. The RSImod, IES, and ERFD are greatly dependent on temporal factors. To explain this matter further, the RSImod is dependent on jump height and total jump time, IES on peak force and the time needed to attain it, and the ERFD on change in force production and time elapsed from the unloading end to the braking end portion of the countermovement (Fig. 5). The RSImod is a valuable measure for assessing explosiveness in athletes^31^. RSImod was originally developed for the drop jump, it has been modified for the CMJ. It is rather practical because drop jumps are technically more demanding; therefore, the values could be unreliable for athletes without extensive experience in such performances.

Moreover, PAPE could have an additional effect on the ERFD. The effect of PAPE could be beneficial by increasing the ERFD, which could hence the acceleration attained with no additional loads and high-velocity movement patterns such as vertical jumps^32^. Furthermore, IES is a valuable parameter used to describe the ERFD minutely. IES expresses the index of explosive strength relative to body mass and is highly correlated with jumping performance^33^.

All parameters mentioned above depend on temporal factors, implying that when an athlete performs a specific jumping movement in a shorter time interval, it significantly affects the overall performance. Therefore, we could assume that PAPE is responsible for acute improvements in the aforementioned kinetic parameters on a force plate. Swimmers faster at 15 m can produce greater velocities and forces on the block than slower swimmers^34^. These findings correspond to the results of a more recent study by Tor et al.^4^, where significant differences and large effects were found in entry velocity between elite male and female swimmers (0.35 m s^−1^, p < 0.001). Male swimmers could generate more force on the starting block and produce higher flight and entry velocities than female ones. The results of our study indicate that the entry time was significantly reduced, especially after the swim-specific warm-up protocol with PAPE, where the effects of the applied protocol were large (see Fig. 1).

If the improvements in the measured kinetic parameters are directly dependent on PAPE, we can assume that the applied protocol could benefit swim start efficiency. Our study found that although drop jumps are not specific to the swim start performance, enhanced neuromuscular potentiation proved beneficial. However, we should note that advanced kinematic and kinetic analyses specific to the swim start have not been performed in this study; therefore, future studies should clarify these findings. In addition, all these parameters have high intraclass correlations and low typical errors and can be valuable measures in performance and training efficiency monitoring. Therefore, apart from JH and PP, other valuable parameters should be considered when assessing jumping performance in general. However, more sophisticated equipment is required to acquire these measurements, which could be a possible limitation for practical purposes.

The practical implications of this study could be reflected in the simplicity of the protocol and its reproducibility, especially in athletes who have little or no experience in strength training. Several studies employed resistance protocols to induce PAPE in swimmers^10–17^ and reported different outcomes; however, we believe that the practical implications of this approach, due to the organisational and schedule limitations of competition, could be time-consuming and somewhat impractical. In addition, not all facilities have the necessary capacity and resources to implement such additional warm-up protocols. Moreover, fatigue should not be neglected during resistance protocols, which could limit more beneficial increments in performance. A recent study suggests that resistance protocols benefit the swim start performance but could affect subsequent phases of a 50-m race due to accumulated fatigue^12^. Few studies^12,16^ reported that the rest period after a resistance protocol should be longer than 6 min to maximise performance. Conversely, some authors stated that 6 min might be enough for performance improvement^15^. However, Seitz et al.^20^ reported that rest intervals after heavy resistance protocols for individuals without extensive experience in heavy resistance training could be prolonged up to 9 min or more, affecting the overall performance. In a recent review^18^, the authors emphasised that swimmers may have to wait up to 20 min between the warm-up and the actual event during competitions. Due to some restrictions in call rooms, this could be a practical issue in pre-competition preparation strategies employing resistance PAPE protocols.

Moreover, the effects of PAPE dissipate after 16 min^19^; therefore, the application of resistance protocols has practical limitations. Conversely, the expediency of drop jumps in PAPE could be justified towards competitors without previous experience in strength training because the potentiation itself caused by resistance protocols is less significant in individuals with undeveloped or less developed strength qualities^21^. The explanation for the previous statement could be that stronger individuals have a significantly higher percentage of type II muscle fibres and could exhibit a greater magnitude of voluntary muscle force enhancement^6,21,35^.

To the best of our knowledge, several credible studies confirmed the effectiveness of drop jumps on PAPE^23–26,30^, while only one study included swimming^28^. A recent meta-analysis reported that plyometrics could induce a slightly more significant stimulus than traditional resistance training^21^. The advantage of the plyometric approach is justified because it produces an effective stimulus somewhat faster than traditional resistance exercises (0.3–4 min, and ≥ 5 min, respectively) and, therefore, could be applied only a few minutes before competition^21^. Chen et al.^24^ reported that recovery should not be more than 6 min because the dissipation of the PAPE stimulus could occur. Therefore, drop jumps could be a valuable tool in addition to a swim-specific warm-up, mainly because they are time-efficient and can be performed just before an event. In addition, the equipment is inexpensive and does not require much space. However, not all the athletes benefitted greatly from the drop jump protocol presented in our study (Figs. 2 and 4). A possible explanation could be found in the drop height assessment. Parameters such as RSI were not considered in the present study, which could accurately determine the ideal stimulus for each individual. In this way, the overall effectiveness of the applied experimental protocols could be altered even further. Moreover, it is possible that three sets of 5 jumps, just a few minutes after a swim-specific warm-up, caused fatigue in some swimmers^24^. Future studies should examine the long-term effects of the drop jump training program on swimmers' performance because PAPE could be maximised with efficient utilisation of the stretch–shortening cycle.

Finally, although we did not examine the mechanisms of PAPE^6–9^ in this study, its mechanistic nature is worth mentioning. It is assumed that there are several mechanisms related to PAPE. In a recent narrative review^6^, authors argue that muscle temperature, muscle blood flow and water content, increased muscle activation, and increased muscle–tendon stiffness could underpin the effects of PAPE., After intense bouts of muscle work, increased muscle temperature could be associated with performance improvements. Based on potential changes in muscle temperature, power output could increase by 1–5%, which is obtained in our study by the power output improvements (3.15 and 1.15%, respectively). Moreover, our study's significant improvements in the RFD could support the assumption that increased muscle temperature could greatly benefit that ability^6^. Increased muscle temperature and blood flow have a similar time course as fluid shifts, increasing force production and possibly explaining PAPE.

Furthermore, based on some evidence, neural changes (increased muscle activation) may be triggered by voluntary activity^36^. However, insufficient evidence suggests that increased muscle activation could not fully explain significant improvements in compound voluntary muscular performance^6^. In addition, from a mechanistic point of view, it has been hypothesised that PAPE increases muscle–tendon stiffness, which could be explained by the fact that peak twitch and the RFD are strongly influenced by the stiffness of intramuscular elastic structures^37^. However, some authors argue that acute alterations in muscle tissue are almost entirely dependent on the changes within the muscle tissue itself^6^. Therefore, a plausible explanation for PAPE could be that increased muscle blood flow and water content trigger the radial bulging of muscles, inducing fibres to rotate further during contraction and generate a passive fibre shortening force^6^. Based on previous statements, we could assume that these mechanisms also apply to ballistic movements such as drop jumps, which can support the justification of incorporating drop jumps as an effective tool for inducing PAPE. However, mechanisms underpinning PAPE have still not been fully explored; therefore, further studies are warranted to clarify this matter. Moreover, as previously stated, our study did not explore the underlying mechanisms regarding PAPE and any decisive conclusion on that matter would be highly speculative.

The following limitations can be pointed out for this study: (1) there was no advanced analysis of PAPE; (2) the effects of different drop jump protocols were not evaluated (more/fewer sets or repetitions); (3) adaptation of a force plate on the swimming starting block to measure kinetic parameters through a specific movement was not possible due to a lack of sophisticated equipment; (4) the latency period between the SW and PAPE protocol to asses accumulated fatigue was not explored. Nonetheless, PAPE induced by drop jumps can positively affect improvements in swim start performance; therefore, there is justification for implementing such a warm-up protocol in practice.

In conclusion, the current findings of this study indicate that the drop jump protocol, in addition to the swim-specific warm-up, is an effective tool due to the athlete's capacity for a more efficient swim start performance. Furthermore, the results of this study indicate that PAPE caused by a drop jump could be time-efficient, economical, and practically applicable in facilities with limited resources. However, more research on this matter is required. Recovery time and prolonged effects following PAPE induced by a drop jump protocol should be more thoroughly investigated since there is no clear consensus. In addition, future research could also consider using the individualised warm-up protocol and recovery periods rather than using common recommendations.

## Methods

### Experimental design

The swimmers performed a swim start at 15 m after each warm-up protocol. The control protocol consisted of a swim-specific warm-up (SW). One experimental protocol included only post-activation performance enhancement (PAPE). Another experimental protocol consisted of a swim-specific warm-up, followed by post-activation performance enhancement (SW + PAPE). In the experimental protocols, the swim start was preceded by PAPE induced by performing three series of 5 drop jumps, while the controls performed the start without PAPE.

### Participants

The sample for this study was composed of ten national-level male swimmers (age, 16 ± 2 years; stature, 1.75 ± 0.07 m; body mass, 64.33 ± 6.08 kg). All of the participants had at least five years of competition experience. The participants underwent eight training sessions per week, up to 2 h per session. General swim-specific conditioning and instructional training were performed in the swimming pool. Dry-land conditioning consisting of bodyweight exercises (squats, lunges, push-ups, and bilateral jumps) was performed twice a week for 30 min. All of the participants had no previous experience in heavy-resistance training. The participants were familiar with the testing protocol, and at the time of the experiment, they were healthy and without any injury. The experimental procedures reported in this study were performed following the ethical standards of the Declaration of Helsinki, and the participants and their parents signed informed consent forms. The experimental protocols were approved by the Ethics Committee of the Faculty of Sport and Physical Education, University of Niš, Serbia.

### Experimental procedures

#### Swim-specific warm-up (SW)

The total volume of SW was set at 1,600 m. The SW consisted of swimming 400 m free/back light swim (75 m freestyle/25 m backstroke), 2 × 100 m individual medley (25 m fly/25 m back/25 m breast/25 m free) with 20 s rest in between, 200 m of front-crawl drills (25 m easy/25 m fast), 200 m of front-crawl kick using a kickboard (25 m fast/25 m easy), 4 × 50 m front-crawl for 60 s (2 easy and 2 medium), 4 × 50 m front-crawl using a starting block (a dive followed by 15 m fast/35 m easy) for 90 s, and a 200 m easy swim using fins. A pre-competition warm-up is an integral part of every competition, typically an active pool-based warm-up that represents preparation for competitive events and enhances the performance of the athletes^38,39^.

#### Drop jump protocol (PAPE)

The drop jump (DJ) was performed from an individualised box height based on the participants' previously evaluated physical ability (40 cm). The participants performed three sets of 5 repetitions based on the recommendations of a previous study^30^. The recovery between repetitions was not longer than 15 s, while the break between sets was 120 s. Drop jumps were performed bouncingly to minimise the ground contact time^40^. It was suggested to the participants that they employ explosive arm swings to maximise their drop jump performance.

Before the experiment, and regardless of the protocol, it was necessary to conduct familiarisation, determine continuity in the CMJ performance on the force plate, and exclude the influence of discontinuous performance on the research results. The first measurement was conducted after a general warm-up. To ensure that the effects of the PAPE came directly from the DJ and not from any other protocol, the swimmers performed 3 CMJs, each separated by 60 s. The intraclass correlation coefficients for test–retest reliability and typical errors for the JH, PP, RSImod, ERFD, and IES were 0.94, 0.96, 0.90, 0.98, and 0.99, and 2.76, 2.66, 0.04, 5.16, and 4.56, respectively. After 48 h of recovery, the second measurement took place. The intraclass correlation coefficients for test–retest reliability and typical error for the swim start to 15 m were 0.97 and 0.38, respectively.

The third, fourth, and fifth measurements were the actual experimental measurements. The recovery between different warm-up protocols was not shorter than 48 h to allow adequate recovery. Moreover, the participants did not undergo intensive training 48 h prior to the experiment, nor did they consume cigarettes, alcohol, or stimulants. Warm-up protocols were implemented in the following order: SW, PAPE, and SW + PAPE. Testing procedures were always performed at the same time of day to exclude diurnal changes in performance. In addition, testing was performed under the same conditions (temperature, humidity, equipment). In the SW + PAPE protocol, the participants performed PAPE 8 min after completing the SW. Instead of SW, the PAPE protocol included a general warm-up (10 min of light skipping, dynamic stretching, and general movement). The participants rested passively for 8 min after the protocols and performed the T15m test^10,17^.

### Measurements

#### Swim start to 15 m

On three separate days, divided by 48 h of recovery, the swimmers performed a 15-m swim start test, preceded by the previously explained warm-up protocols. Track start was adopted through all conditions. The participants were instructed to perform a maximum effort dive, underwater kick, and freestyle swim to the 15-m mark. The starting block specifications were as follows: the height above the water surface was 0.72 m from the water surface, with a 0.5 × 0.5 m platform and an 8° inclination. The Alge Swim Time platform (SO2-X, Alge Timing—Austria) that measures the start reaction was placed on the start block platform. Time was measured in seconds. Before performing each start, the participant was instructed, by a verbal command, to a position on the starting block. For better efficiency, the participants performed the swimming start with maximum freestyle intensity, even a few meters after the 15-m mark.

To collect the parameters of the swim start, we used two digital cameras. A CASIO FX camera that records 300 frames per second was used for the kinematic analysis of the swim start. The camera was placed in the sagittal plane and perpendicular to starting lane 8. Moreover, the camera was static and adjusted to the horizontal optical axis, approximately 1 m in front of the vertical plane of the leading edge of the starting block and 1 m above the water surface. The second, a Canon RT digital video camera, which records 60 frames per second, was placed perpendicular to the 15-m line from the starting block, and its role was to record the moment the participant's head passed through the 15-m mark. Both cameras were recording continuously during each testing protocol. The cameras were set up to record visual and audio signals. Alge Timing emits the audio start signal and a simultaneous visual flash from a strobe placed opposite the cameras.

Kinematic analysis of the swim start was performed using computer software for 2D kinematic analysis (Dartfish, v. 4.5.2.0, Fribourg, Switzerland).

The measured parameters for the swim start performance were time at 15 m (T15m, time from the start signal until the moment when the swimmer's head passes through the 15-m mark) and entry time (ET, time from the first contact with the water to full body entry).

#### Countermovement jump

The CMJ was used to measure the power of the lower extremities, with a force plate (Kistler, QuattroJump 9290DD, Winterthur, Switzerland), with a sampling rate of 500 Hz. Before performing the jump, the participants were instructed to put their hands on their hips. The CMJ was performed at the swimming pool, in a swimsuit and barefoot to bring the measurement procedure closer to actual conditions and achieve good ecological validity.

Before performing the CMJ, the participants stood motionless on a force plate (Kistler QuattroJump) for more than one second to measure their body weight (N)^41^. Raw ground reaction force (GRF) data were exported for processing into custom adapted software (MATLAB, v. R2018a (9.4.0.813654), MathWorks, Inc., Natick, Massachusetts, USA). Vertical GRF data were extracted only along the vertical axis (Fz). After extracting raw Fz from a force plate, the data were smoothed using a fourth-order low pass Butterworth digital filter with a cut-off frequency of 50 Hz^42^. The Fz jump profile was divided into unloading, eccentric (yielding and braking), and concentric phases (Fig. 5). The onset of movement^43^, the onset of braking and the concentric phase^41^, and contact time^43^ were determined with the methods proposed in a recent study.

The measured parameters were jump height (JH), the reactive strength index modified (RSImod), peak power (PP), the eccentric rate of force development (ERFD), and the index of explosive strength (IES). All dependent variable calculations are presented in Table 2.

**Table 2:** Calculation of dependent variables.

Variable	Calculation
Jump height (JH)	(Takeoff velocity)^2^/(2 × 9.81)
Reactive strength index modified (RSImod)	(Jump height)/(Jump time)
Peak power (PP)	Maximum PO during CMJ (Force*Velocity)/(Body mass)
Eccentric RFD (ERFD)	(1^st^ Force _peak_ − minimum Force)/(time)
Index of explosive strength (IES)	(Force _peak_/time to Force_peak_)/(Body mass)
Jump time	(Time at take-off) − (Onset of movement)

### Statistical analysis

All data analyses were carried out using the SPSS Statistical Package for Social Sciences (IBM SPSS, version 23.0; IBM SPSS, Armonk, NY, United States). Means, standard errors, and the Kolmogorov–Smirnov test were computed. Differences were compared by using the one-way repeated-measures ANOVA and the Bonferroni method. Effect size (ES) and percentage of change in performance were also computed. Moreover, the smallest important effect for each dependent variable was applied to observe the actual change in performance. The smallest important effect was defined as the smallest worthwhile change deemed practically significant to the swimmers. For each effect, the threshold value for the smallest important effect was 0.2. The probabilities that the actual difference in performance was harmful, trivial, or beneficial were expressed as percentages, reflecting the following descriptors: 1%, almost certainly not; 1–5%, very unlikely; 5–25%, unlikely; 25– 75%, possibly; 75–95%, likely; 95–99%, very likely; 0.99%, almost certainly^44^.

### Ethics declarations

The Ethics Committee of the Faculty of Sport and Physical Education, University of Niš, Serbia, obtained ethical review and approval. The experimental procedures reported in this study were performed following the ethical standards of the Declaration of Helsinki, and the participants and their parents signed informed consent forms.

## Data Availability

The raw data supporting the conclusions of this article will be made available by the authors without undue reservation.
